# Cerebral perfusion in post-stroke aphasia and its relationship to residual language abilities

**DOI:** 10.1093/braincomms/fcad252

**Published:** 2023-10-05

**Authors:** Maria V Ivanova, Ioannis Pappas, Ben Inglis, Alexis L Pracar, Timothy J Herron, Juliana V Baldo, Andrew S Kayser, Mark D’Esposito, Nina F Dronkers

**Affiliations:** Department of Psychology, University of California, Berkeley, CA 94720, USA; Research Service, VA Northern California Health Care System, Martinez, CA 94553, USA; Stevens Neuroimaging and Informatics Institute, University of Southern California, Los Angeles, CA 90033, USA; Henry H. Wheeler, Jr. Brain Imaging Center, University of California, Berkeley, CA 94720, USA; Department of Psychology, University of California, Berkeley, CA 94720, USA; Research Service, VA Northern California Health Care System, Martinez, CA 94553, USA; Research Service, VA Northern California Health Care System, Martinez, CA 94553, USA; Division of Neurology, San Francisco VA Health Care System, San Francisco, CA 94121, USA; Department of Neurology, University of California San Francisco, San Francisco, CA 94158, USA; Department of Psychology, University of California, Berkeley, CA 94720, USA; Neurology Service, VA Northern California Health Care System, Martinez, CA 94553, USA; Department of Psychology, University of California, Berkeley, CA 94720, USA; Depertment of Neurology, University of California, Davis, CA 95817, USA

**Keywords:** aphasia, language, perfusion, perilesional, temporoparietal areas

## Abstract

Stroke alters blood flow to the brain resulting in damaged tissue and cell death. Moreover, the disruption of cerebral blood flow (perfusion) can be observed in areas surrounding and distal to the lesion. These structurally preserved but suboptimally perfused regions may also affect recovery. Thus, to better understand aphasia recovery, the relationship between cerebral perfusion and language needs to be systematically examined. In the current study, we aimed to evaluate (i) how stroke affects perfusion outside of lesioned areas in chronic aphasia and (ii) how perfusion in specific cortical areas and perilesional tissue relates to language outcomes in aphasia. We analysed perfusion data from a large sample of participants with chronic aphasia due to left hemisphere stroke (*n* = 43) and age-matched healthy controls (*n* = 25). We used anatomically defined regions of interest that covered the frontal, parietal, and temporal areas of the perisylvian cortex in both hemispheres, areas typically known to support language, along with several control regions not implicated in language processing. For the aphasia group, we also looked at three regions of interest in the perilesional tissue. We compared perfusion levels between the two groups and investigated the relationship between perfusion levels and language subtest scores while controlling for demographic and lesion variables. First, we observed that perfusion levels outside the lesioned areas were significantly reduced in frontal and parietal regions in the left hemisphere in people with aphasia compared to the control group, while no differences were observed for the right hemisphere regions. Second, we found that perfusion in the left temporal lobe (and most strongly in the posterior part of both superior and middle temporal gyri) and inferior parietal areas (supramarginal gyrus) was significantly related to residual expressive and receptive language abilities. In contrast, perfusion in the frontal regions did not show such a relationship; no relationship with language was also observed for perfusion levels in control areas and all right hemisphere regions. Third, perilesional perfusion was only marginally related to language production abilities. Cumulatively, the current findings demonstrate that blood flow is reduced beyond the lesion site in chronic aphasia and that hypoperfused neural tissue in critical temporoparietal language areas has a negative impact on behavioural outcomes. These results, using perfusion imaging, underscore the critical and general role that left hemisphere posterior temporal regions play in various expressive and receptive language abilities. Overall, the study highlights the importance of exploring perfusion measures in stroke.

## Teaser

Ivanova *et al*. using perfusion MRI demonstrate that blood flow in chronic post-stroke aphasia is significantly reduced beyond the lesion site compared to age-matched controls and that hypoperfusion in critical temporoparietal areas has a negative impact on language outcomes. The study highlights the importance of exploring perfusion measures in stroke.

## Graphical Abstract

**Graphical Abstract:**
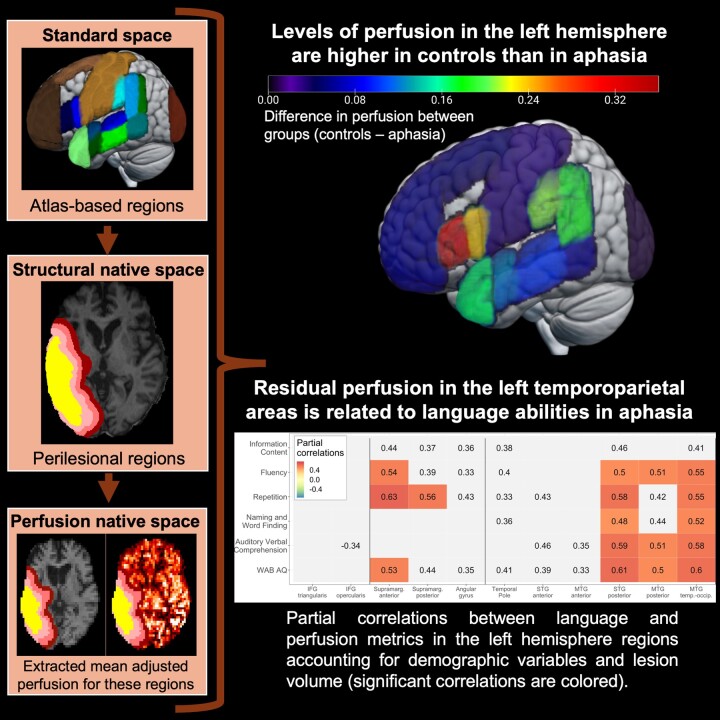

See Thompson and Walenski (https://doi.org/10.1093/braincomms/fcad341) for a scientific commentary on this article.

## Introduction

Stroke is a heterogeneous syndrome caused by multiple pathological mechanisms resulting in the disruption of cerebral blood flow (CBF). Decreases in the rate of delivery of blood to brain tissue (measured in units of ml blood/100 g tissue/min) result in subsequent cell death and tissue loss due to the lack of necessary oxygen^1^ leading to motor, cognitive, and language impairments in stroke survivors. In human adults, normal CBF in different grey matter regions typically ranges from 35 to 80 mL/100 g/min depending on various factors such as age, sex, diet, cardiovascular fitness, and health status.^2-5^ CBF is generally considered to be coupled with cerebral glucose metabolism,^6,7^ making it an important indicator of tissue functionality. Specifically, in animal models, it was shown that brain tissue needs to be perfused at 10% of normal levels to survive and at least at 30–50% for neuronal function (i.e. electrical signalling) to continue.^8,9^ Subsequently, it is expected that lower perfusion may potentially impede normal functioning.^10-12^ Specifically in the context of aphasia, it remains to be answered how CBF after stroke in different brain regions relates to language outcomes and whether it can provide additional insights into the mechanisms of post-stroke recovery.

Research from the acute stroke literature using MRI techniques has demonstrated that CBF is typically disrupted and lowered in perilesional tissue, areas surrounding the neural regions that are permanently damaged.^13-15^ These aberrant perfusion effects persist in the chronic phase, with perilesional perfusion reduced relative to homologous areas in the contralesional hemisphere and similar regions in age-matched healthy controls.^16-20^ The relationship of hypoperfusion of perilesional tissue to language outcomes has been mixed. Some studies have shown that levels of perilesional perfusion are associated with cognitive and language impairments^16,18,21,22^ and recovery of motor function,^23^ while others failed to find systematic association between perilesional perfusion and language outcomes.^17,24^

Disruption of perfusion in stroke can also be found beyond perilesional areas. Decreased CBF has been observed in ipsilateral areas distant to the lesion.^5,19,25^ A recent comprehensive study of perfusion in chronic aphasia showed a decrease in perfusion in several areas in the left hemisphere compared to healthy controls [and somewhat unexpectedly an increase in the superior frontal gyrus (SFG)]; however, these differences were not associated with functional language outcomes.^16^ In this study, the left hemisphere regions of interest (ROIs) falling within the distribution of the middle cerebral artery were hypoperfused, while regions in the anterior cerebral artery were hyperperfused. Another recent small cohort study employing individualized perfusion cut-offs based on right hemisphere perfusion values demonstrated a strong relationship between hypoperfusion of regions in the left posterior temporal and inferior parietal areas and general language ability and auditory comprehension.^5^ Furthermore, perfusion levels in the middle cerebral artery territory of the left hemisphere have been shown to predict stroke recovery, with initially higher CBF predictive of better treatment outcomes in aphasia.^17,24,26^

A few studies that have investigated the effects of perfusion in the right contralateral hemisphere have found increased perfusion.^16,17,25^ Thompson *et al.*^16^ proposed that perfusion in the right hemisphere is increased potentially as a form of compensatory autoregulatory process, on the assumption that total blood flow to the brain remains approximately the same post-stroke. It is unclear whether these changes are related to functional outcomes. Thompson *et al.*^16^ found no such relationship. Conversely, Boukrina *et al.*^17^ showed that increased perfusion in the right hemisphere regions that are part of the reading network based on the Neurosynth database^27^ was associated with lower word reading accuracy both in the subacute and the chronic stages (but not related to performance on other language tasks).

The lack of consensus in prior perfusion studies in aphasia may be due to small sample sizes (typically 1–30 participants) and other methodological issues, such as age differences between the aphasia and the control groups, different imaging sequences and processing algorithms, arbitrary cut-offs, varying definitions of perilesional areas, and different parcellations. Larger group studies using perfusion in chronic post-stroke aphasia are needed to address existing knowledge gaps.

In the current study, we aimed to explore how stroke affects perfusion outside of lesioned areas in chronic post-stroke aphasia and how perfusion in specific cortical areas and perilesional tissue relates to language outcomes in aphasia. Specifically, we hypothesized that perfusion in stroke patients would be reduced outside of lesioned areas, both in perilesional and distant areas, compared to the right hemisphere and to perfusion in age-matched healthy controls. Also, we hypothesized that perfusion levels specifically in perisylvian areas, regions typically known to support language, but not in other brain areas would correlate with language abilities in post-stroke aphasia. Our current work expands upon previous studies in several important ways. First, we analyse one of the largest samples to date of chronic patients with aphasia scanned with perfusion imaging. Second, we explore regions in both hemispheres as well as systematically investigate perfusion in perilesional areas, examining separately perilesional tissue at varying distances from the lesion. Finally, we explore the contribution of perfusion to language abilities in multiple regions simultaneously while accounting for structural damage.

## Materials and methods

### Participants

Forty-three participants with aphasia (PWA; 29 males and 14 females) following a left hemisphere stroke (*M*_age_ = 65.5 ± 11.1 years, from 43 to 88 years of age) were recruited for the study. All participants except three were strongly right-handed based on the Edinburgh Handedness Inventory,^28^ with these three participants reporting a right-hand preference but some ambidexterity. All passed screening tests for any hearing and visual deficits and had native-like proficiency in English prior to their stroke. All participants had suffered a single stroke, except for small (<2 cm) asymptomatic secondary events, with the most recent incident being at least 3 months prior to testing and scanning (*M*_time post-onset_ = 52.2 ± 72.3 months). PWA in this sample presented with a wide range of speech and language deficits, some performing within normal limits on the Western Aphasia Battery (WAB^29,30^) but still complaining of residual naming and/or comprehension deficits (see Results section for more information). All patients signed Institutional Review Board-approved consent forms and were tested in accordance with the Helsinki Declaration.

A group of healthy age-matched controls was also recruited. Twenty-five participants (19 males, 6 females) with no neurological history participated (*M*_age_ = 61.6 ± 11.3 years, from 41 to 84 years of age). There was no significant difference between the control and aphasia groups in age [*t*(66) = −1.35, *P* = 0.18] or gender distribution [*χ*^2^(1, *N* = 68) = 0.56, *P* = 0.46].

### Behavioural assessments

The WAB^29,30^ was administered to evaluate the language abilities of the PWA. Participants were assessed with the 10 main language subtests, which contribute to the following subtest scores: fluency, information content, repetition, naming, and auditory comprehension. Scores from these subtests comprise the WAB aphasia quotient (AQ), a general measure of aphasia severity.

### MRI data: acquisition, pre-processing, and perfusion data analysis

Structural MRI (T_1_-weighted) and perfusion data were acquired. The participants were scanned at two different sites [Veterans Affairs (VA) and University of California Berkeley (UCB)] with slightly differing protocols described below. To account for possible interscanner variations, we normalized individual perfusion by whole-brain perfusion and used scanning site as a covariate in all the correlation/regression analyses (see section Statistical analysis).

### Data acquisition

#### VA cohort

VA participants underwent anatomical and ASL scans on a 3 T Siemens Verio scanner with a 12-channel phased-array head coil at the VA Hospital in Martinez, CA. For perfusion, a pseudo-continuous arterial spin labelling sequence was used with the following parameters: TR/TE = 4000/12 ms, flip angle = 90°, bandwidth = 2.6 KHz/pixel, Field of View (FOV) = 22 cm, voxel size = 3.4 × 3.4 × 6 mm, slice-selective gradient = 6mT/m, and 20 axial slices in ascending sequential acquisition order using echo planar imaging readout. The labelling duration was 1470 ms with a post-labelling delay of 1500 ms. A total of 80 images were acquired in the interleaved tag/control order for each subject. Since a separate M0 calibration image was not obtained, per current recommendations,^31^ we used the first control image as a calibration image for the analysis of this cohort. One high-resolution anatomical image was acquired for each subject with the scan parameters: MP-RAGE sequence, TR/TE = 2200/1.62 ms, TI = 900 ms, flip angle = 9°, FOV = 256 mm, voxel size 1 × 1 × 1 mm, 192 sagittal slices, bandwidth = 340 Hz/voxel, and GRAPPA factor = 2. Twenty-nine PWA and all 25 controls were scanned at this location.

#### UCB cohort

UCB participants underwent anatomical and ASL scans on a Siemens 3 T Trio scanner with a 32-channel coil at the Henry H. Wheeler Jr. Brain Imaging Center, UCB, CA. For perfusion imaging, a pseudo-continuous arterial spin labelling sequence with spiral readout was used with the following parameters: TR/TE = 4600/8.7 ms, flip angle = 90°, bandwidth = 400 Hz/Pixel, FOV = 25 cm, 40 slices, voxel size = 3 × 3 × 3 mm, and phase encoding gradient = 6 mT/m. The labelling duration was 1800 ms with a post-labelling delay of 2000 ms. A total of 16 images were acquired in the interleaved tag/control order for each subject. A stack-of-spirals readout was used with a four-shot spiral interleave for each of the 3D phase encoding steps with background suppression on.^32^ An equilibrium magnetization (M0) image was also obtained and later used in the kinetic model to compute CBF values. One high-resolution anatomical image was also acquired for each subject with the following parameters: MP-RAGE sequence, TR/TE = 2300/2.96 ms, TI = 900 ms, flip angle = 9°, FOV = 256 mm, voxel size 1 × 1 × 1 mm, 208 sagittal slices, bandwidth = 240 Hz/voxel, and GRAPPA factor = 2. Fourteen PWA were scanned at this location.

### ASL data pre-processing

The processing steps were identical for the two cohorts. Tag-control images were motion corrected using FMRIB Software Library (FSL) function MCFLIRT.^33^ CBF maps were obtained using FSL’s command ‘oxford_asl’ on the motion corrected ASL data^34^ with the parameters tailored to reflect each site’s acquisition parameters. CBF maps were quantified in standard physiological units (ml blood/100 mg tissue/min) using a standard kinetic model.^31^ Labelling efficiency was set to *α* = 0.72, and the longitudinal relaxation time of the blood was set to T_1__b = 1650 ms. No further smoothing was performed. It is worth noting that partial volume correction methods were not applied to the data due to the presence of the lesions and the lack of consensus on the best partial volume correction method that can be applied to ASL data.^35^ We plan to systematically investigate the effect of partial volume correction on ASL in stroke in the future.

### Lesion segmentation and structural data pre-processing

**Figure 1:**
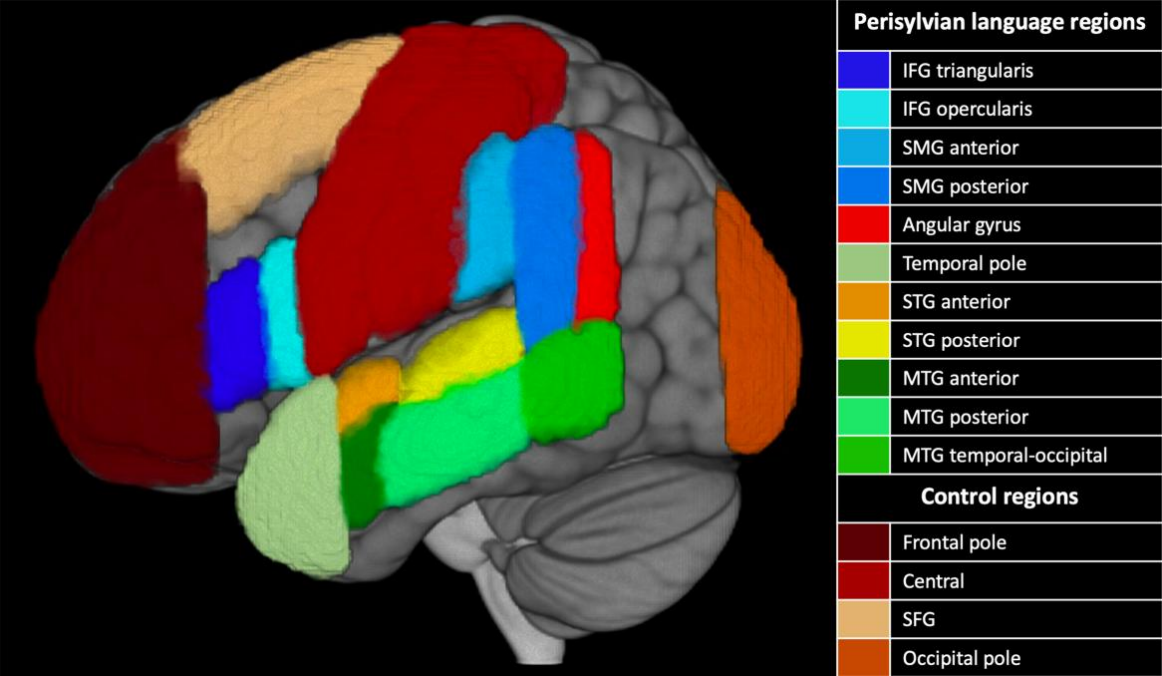
**Representation of atlas-based ROIs taken from the Harvard–Oxford atlas.** ^39^

The participants’ lesions were traced directly onto the patient’s native T_1_-weighted images using MRIcron software^36^ by trained research assistants and then reviewed and verified by Maria V. Ivanova, Ioannis Pappas, and Nina F. Dronkers. Next, we used the Advanced Normalization Tools (ANTs)^37^ to perform brain extraction on the structural T_1_s. We also used ANTs to segment the T_1_ images and obtain probability maps corresponding to different tissues (grey matter, white matter, and cerebrospinal fluid). We then performed the following registrations to be able to bring ROIs to native CBF space. (**A**) The CBF maps were registered to the brain extracted images (T_1_ native space) using an affine transformation. (**B**) The brain-extracted T_1_ images were registered to the MNI-152 space. To do so, we used ANTs with a transformation that consists of an initial rigid plus affine transformation followed by a diffeomorphic, ‘SyN’ transformation, while cost function masking the lesion. We used the MNI152NLin2009cAsym version of the MNI image as the target MNI image.^38^ Using the inverse of the transformation obtained in (**A**), we were able to map individually defined lesion masks, as well as perilesional ROIs, from native T_1_ space to CBF space. Using the combined inverse transformations of (**A**) and (**B**), we were able to map atlas-based ROIs defined in MNI-152 to native T_1_ and then to CBF space. In the analysis, we use the following ROIs:

- *Atlas-based ROIs* were taken from the Harvard–Oxford Cortical Structural Atlas in FSL.^33,39^ The segmentation from this atlas was selected as it contains ROIs of optimal size. Given the low resolution of the perfusion data, we needed ROIs large enough to minimize partial volume effects but small enough to ensure adequate spatial specificity for distinct language regions. From the atlas, we selected 11 ROIs that covered Perisylvian language regions [inferior frontal gyrus (IFG) triangularis, IFG opercularis, supramarginal gyrus (SMG) anterior, SMG posterior, angular gyrus, temporal pole, superior temporal gyrus (STG) anterior, STG posterior, middle temporal gyrus (MTG) anterior, MTG posterior, and MTG temporal–occipital]. We were interested whether perfusion levels in those ROIs would be related to residual language abilities. We also included four control ROIs (frontal pole, central, SFG, and occipital pole) that covered regions typically not related to language processing. See Fig. 1 for representation of these ROIs. These ROIs in MNI-152 space were brought to native CBF space using the combination of transformations described previously in (**A**) and (**B**).
- *Perilesional ROIs* were obtained by expanding the lesion mask in native T_1_ space by 5 mm and subtracting the original lesion mask from it. This process was repeated stepwise to obtain additional perilesional masks for 5–10 and 10–15 mm bands outside the lesion. These three perilesional ROIs were brought to CBF space using the inverse affine transformation described previously in (**A**).

### ROI analysis of ASL data

For all the ROIs, mean CBF values were obtained as the average CBF within each ROI. Because some ROIs overlapped with regions of no interest (e.g. perilesional ROIs could end up covering the ventricles or include voxels outside the brain), we required the CBF values of each ROI to exclude any cerebrospinal fluid values or values outside the brain mask for all our calculations. Right hemisphere areas were also excluded from all perilesional ROIs. In addition, for the PWA cohort, lesioned voxels determined by the lesion mask were excluded from all the atlas-based ROIs at the participant level. Finally, lesion load was defined as the percentage of voxels in the ROI covered by the lesion.

### Statistical analysis

For all the statistical analyses in both the PWA and the control groups, we divided perfusion values by the whole-brain perfusion signal (mean perfusion across the whole brain with the lesion site masked for the PWA group). This approach allowed us to account for individual variability in tagging efficiency and other potential session-specific and scanner-specific artefacts. To control for multiple comparisons, we adjusted the *P*-value in each analysis by the number of atlas-based ROIs in each hemisphere. Thus, our critical significance threshold was *P* < 0.0033 (0.05/15; two-tailed) for both between-group comparisons and correlation analyses. All data analyses were performed in R ver. 4.1.2.,^40^ and figures were drawn in ggplot2 ver. 3.3.5.^41^

For between-group comparisons of perfusion levels in different ROIs, the following procedure was implemented. First, we checked whether perfusion data for a specific ROI for each group followed a normal distribution (Shapiro test). If the data were normally distributed in both groups for that ROI, we ran an *F*-test to test for homogeneity of variances. For those ROIs where all these assumptions were satisfied, we ran the standard independent samples *t*-tests to compare perfusion levels between groups. If the assumption of homogeneity of variances was violated, an unequal variances *t*-test (Welch’s test) was performed. If in both or in one of the groups the data were not normally distributed, then the non-parametric two-sample Wilcoxon rank test was performed. This procedure was implemented for comparing PWA to age-matched controls and for checking whether perfusion levels differed between those PWA with a lesion in a given atlas-based ROI and those without that lesion. Also, to explore the relationship between perfusion levels and lesion status, we performed correlations between the two metrics—mean adjusted perfusion and lesion load—for each ROI.

For within-group comparisons of adjusted perfusion levels in left hemisphere ROIs to homologous right hemisphere ROIs, we first verified that the differences between homologous left and right ROIs were normally distributed. If that assumption was satisfied, then a standard paired sample *t*-test was run. If not, then we performed a paired two-sample Wilcoxon test.

Finally, to analyse the relationship between language measures and perfusion levels, we first performed a partial correlation analysis between mean adjusted perfusion in an ROI and language metrics, accounting for age, gender, time post-onset, scanning site, and lesion volume. In addition to using the omnibus lesion volume, we accounted for lesion load to that specific ROI as well. Partial correlation analysis was done with the package ppcor.^42^ This analysis was followed by a regularized lasso regression, so that we could (i) analyse the impact of perfusion in all the ROIs simultaneously, while controlling for lesion load to the respective ROIs, and (ii) outline in which regions perfusion levels were associated with different language abilities. Regularized regression allows determination of salient relationships in complex data sets and has been successfully used previously in neuroimaging studies to select relevant neural predictors.^43^

Lasso regression (*α* = 1) differs from traditional multiple linear regression as it employs L1-normalization to regularize model coefficients so that unimportant features are eliminated, preventing overfitting and improving generalization on test data.^44,45^ This approach is also recommended for instances when the number of cases is comparable to the number of predictors, as in the present case. The regularization term provides a constraint on the size of weights, and it is controlled by parameter *λ*, with larger *λ* leading to more shrinkage. When there are many correlated variables in an ordinary linear regression model, the weights can be poorly determined and exhibit high variance. Using *λ* to impose a size constraint on the coefficients alleviates this problem, and a lasso (L1) regularized regression specifically will assign beta weights of 0 to weak predictors.^44^ In this analysis, we included all the covariates from the partial correlation above and all the perilesional and atlas-based ROIs. Predictors first had to be standardized, so that their absolute values would not influence the weights. Next, an optimal *λ* was selected through leave-one-out cross-validation, with the *λ* that minimized residual mean squared error in the model used in the analysis. These regression analyses were performed with the glmnet package.^46^

## Results

### Differences in perfusion levels between groups

**Figure 2:**
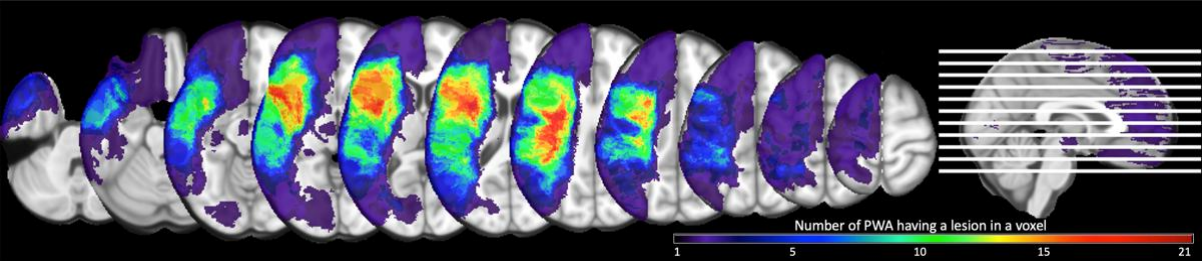
**Lesion overlay map.** The map shows overlap across participants’ lesions (*n* = 43), with brighter colours indicating greater number of participants with aphasia having a lesion in each voxel (ranging from a minimum of one participant’s lesion in a voxel to a maximum of 21).

Lesion overlays for the aphasia group are presented in Fig. 2. Maximal lesion overlap was observed in frontal and subcortical temporoparietal areas.

**Figure 3:**
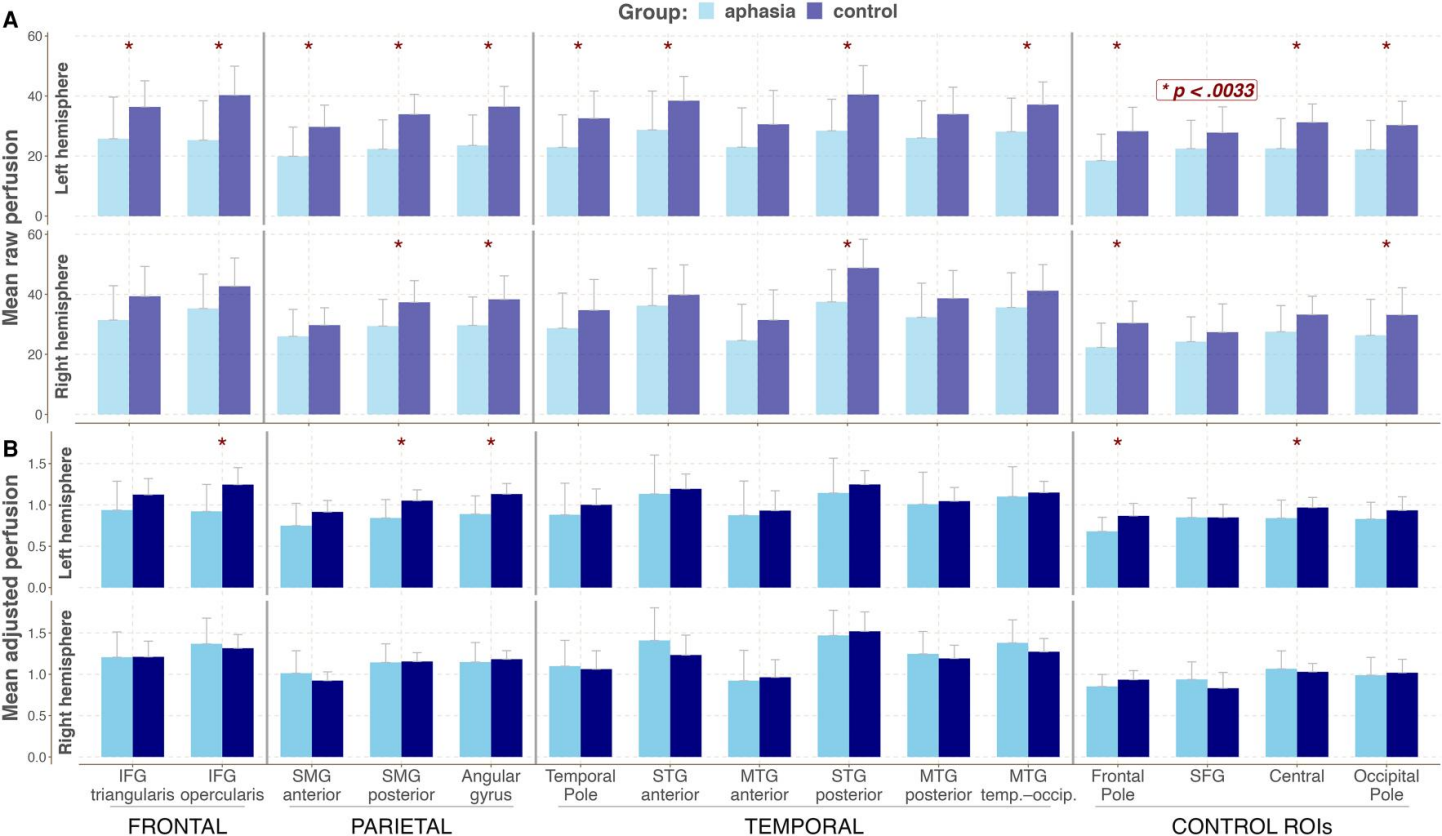
**Perfusion levels across the left and right hemispheres in the aphasia and the control groups.** Mean perfusion values for the aphasia (*n* = 43) and the control (*n* = 25) groups across left and right hemisphere ROIs. Asterisks mark significant differences between groups for a given ROI. (**A**) Mean raw perfusion. (**B**) Mean adjusted perfusion. The statistical comparisons were done using the independent samples *t*-test/Welch’s test/non-parametric two-sample Wilcoxon rank test (see section Statistical analysis for further details). See Supplementary Tables A1 (raw perfusion) and A2 (adjusted perfusion) for actual values and relevant test statistics.

Raw and adjusted (by the whole-brain perfusion) perfusion values for left and right hemisphere ROIs for the aphasia and the control groups are presented in Fig. 3A and B, respectively (see Supplementary Tables A1 and A2 for actual values and test statistics). For this and all subsequent analyses, lesioned voxels were excluded from the calculation of perfusion in the left hemisphere. As can be seen from Fig. 3A, the majority of the left hemisphere ROIs and some of the right hemisphere ROIs showed significantly lower raw perfusion values in PWA relative to controls as determined by independent samples *t*-tests/Wilcoxon rank tests. Whole-brain perfusion was also significantly lower in PWA compared to controls [*M*_Controls_ = 32.4, *M*_PWA_ = 26.2; *t*(66) = 3.42, *P* = 0.001]. For the adjusted perfusion values (Fig. 3B), only left ROIs, specifically regions in the frontal and parietal regions, showed significantly decreased perfusion in the PWA group compared to age-matched controls. No differences were observed in the right hemisphere.

To ensure that the results were not driven by any sequence-specific parameters, we compared raw and adjusted perfusion values for the participants from only the VA cohort. This analysis yielded largely similar findings, indicating between group differences in adjusted perfusion for the left frontal and parietal regions (see Supplementary Fig. B1 for details). Furthermore, no systematic differences could be identified in adjusted perfusion between the VA and the UCB cohorts (see Supplementary Table B1), further justifying combining data from the two scanning sites. For all subsequent analyses, adjusted perfusion values from both cohorts are used.

### Differences within groups: comparing perfusion levels in homologous regions in the left and right hemispheres

**Figure 4:**
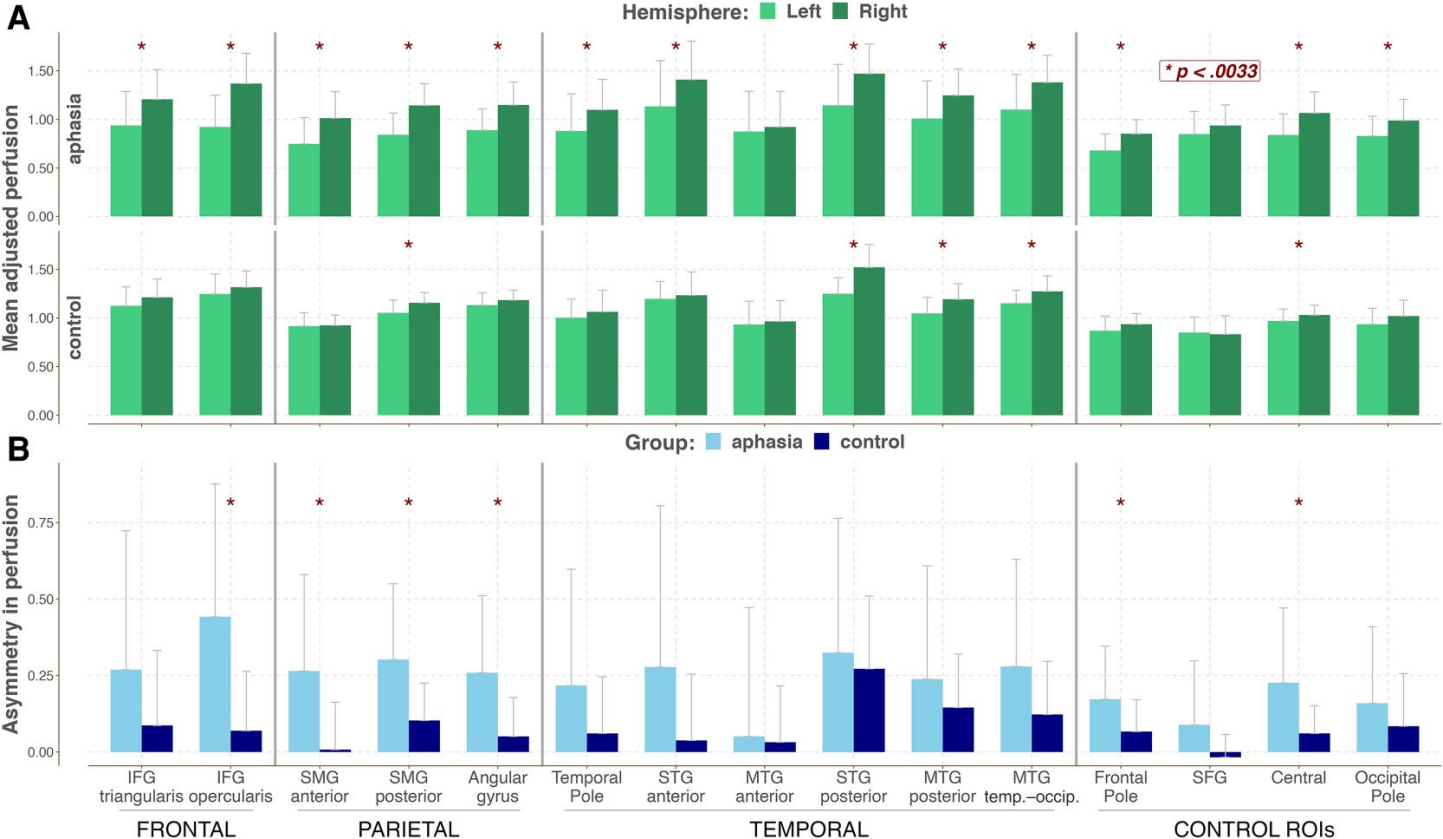
**Comparison of perfusion in the left and right hemisphere ROIs between the two groups.** Asterisks mark significant differences. (**A**) Mean adjusted perfusion for the left and right hemisphere ROIs in the aphasia (*n* = 43) and the control (*n* = 25) groups. (**B**) Asymmetry of right–left hemisphere perfusion between the aphasia (*n* = 43) and the control (*n* = 25) groups. The statistical comparisons in **A** were done using either the paired samples *t*-test or the paired two-sample Wilcoxon test. The statistical comparison in **B** were done using the independent samples *t*-test/Welch’s test/non-parametric two-sample Wilcoxon rank test. See Supplementary Table A3 for relevant test statistics.

Next, we compared perfusion levels in the left hemisphere ROIs to homologous regions in the right hemisphere (see Fig. 4A) for both the control and aphasia groups (see Supplementary Table A3 for test statistics). There was a statistically significant decrease in perfusion across all regions of the left hemisphere in the aphasia group, except the anterior part of the MTG and SFG. The control group showed interhemispheric differences for a limited set of regions, including the posterior portion of the SMG, the posterior part of the temporal lobe, and the area around the central sulcus, with the left ROIs having lower perfusion compared to their right hemisphere counterparts. Additionally, we compared the extent of this asymmetry (differences between perfusion in the right versus left hemispheres) between the two groups (see Fig. 4B). Asymmetry of perfusion was more pronounced in the PWA group compared to the control group for frontal and parietal regions, indicating that the PWA group showed greater differences between perfusion in homologous ROIs specifically in these areas.

### Impact of lesion on perfusion levels

**Figure 5:**
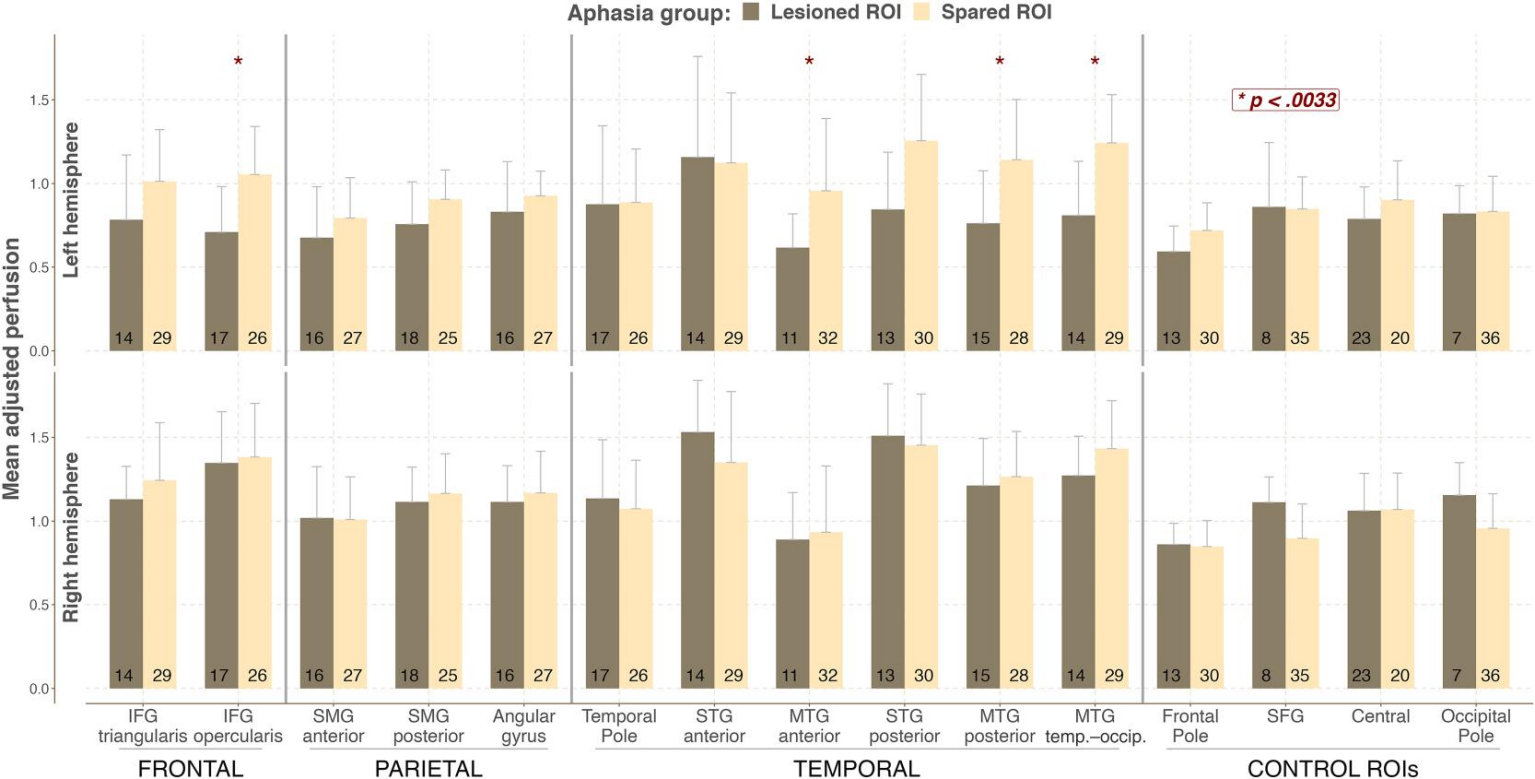
**Differences in perfusion depending on the region’s lesion status.** Mean adjusted perfusion for the left and right hemisphere ROIs in the aphasia group with (‘Lesioned ROI’) and without a lesion (‘Spared ROI’) in a given ROI in the left hemisphere. For each ROI for both hemispheres, participants are grouped into two subgroups—those in whom this ROI was lesioned in the left hemisphere and those in whom it was spared. The number in each column indicates the number of participants in each subgroup. Asterisks mark significant differences between the two aphasia subgroups. The statistical comparisons were done using the independent samples *t*-test/Welch’s test/non-parametric two-sample Wilcoxon rank test. See Supplementary Table A4 for actual values and relevant test statistics.

Next, we investigated whether perfusion levels were impacted by having a lesion in a specific ROI. For each ROI, we split participants into two groups—those in whom this ROI was lesioned in the left hemisphere (lesion load greater than 0) and those in whom it was spared (lesion load equal to 0). We then compared the perfusion levels between these two groups in that specific ROI in both hemispheres. This procedure was repeated across all the ROIs. In other words, for each comparison, we regrouped the participants based on their lesion load for that specific ROI in the left hemisphere. Quantitatively perfusion was higher in the ‘Spared ROI’ group compared to the ‘Lesioned ROI’ group across the left perisylvian regions, but the difference was statistically significant only for the IFG opercularis and different parts of the MTG (see Fig. 5; see Supplementary Table A4 for actual values and test statistics). When the same analysis was performed for homologous right hemisphere ROIs, no significant or systematic differences were observed between the two groups. In other words, a lesion in the left hemisphere had no impact on perfusion in the homologous right hemisphere regions.

Further, we explored the relationship between perfusion levels and lesion location by correlating perfusion levels across the different left hemisphere ROIs with their respective lesion load. Non-parametric spearman correlations were performed as the lesion load data were not normally distributed. Significant correlations were only detected for the IFG opercularis (*r* = −0.57, *P* < 0.001), posterior (*r* = −0.48, *P* = 0.001), and temporal–occipital part of the MTG (*r* = −0.6, *P* < 0.001). These findings indicate that, in these left hemisphere ROIs, having a higher lesion load was associated with having lower perfusion levels in the spared parts of these regions.

Finally, we compared perfusion in the three different perilesional ROIs. Perfusion in the perilesional 0–5 mm band (*M*_0–5mm_ = 0.86 ± 0.19) was significantly lower than in the 5–10 mm band [*M*_5–10mm_ = 0.96 ± 0.16; *t*(42) = −7.5, *P* < 0.001], which in turn was lower than in the 10–15 mm band [*M*_10–15mm_ = 1.01 ± 0.12; *t*(42) = −3.14, *P* = 0.003]. Only perfusion in 0–5 mm band was significantly lower compared to whole-brain perfusion [*t*(42) = −4.9, *P* < 0.001].

### Correlation between language measures and perfusion levels

Mean WAB subtest scores for the PWA group are presented in Table 1. Note that while some participants had WAB AQ scores above the 93.8 cut-off for aphasia, they continued to experience minor language deficits, particularly in word finding.

**Table 1:** Descriptive statistics for WAB subtest scores

	Information content	Fluency	Repetition	Naming	Auditory comprehension	WAB AQ
**Mean (SD)**	8.7 (2)	8 (2.5)	7.9 (2.8)	7.6 (2.7)	9 (1.3)	82.3 (20.1)
**Range (min—max)**	3–10	0–10	0–10	0–10	5.4–10	22.8–100
**Max score possible**	10	10	10	10	10	100

**Figure 6:**
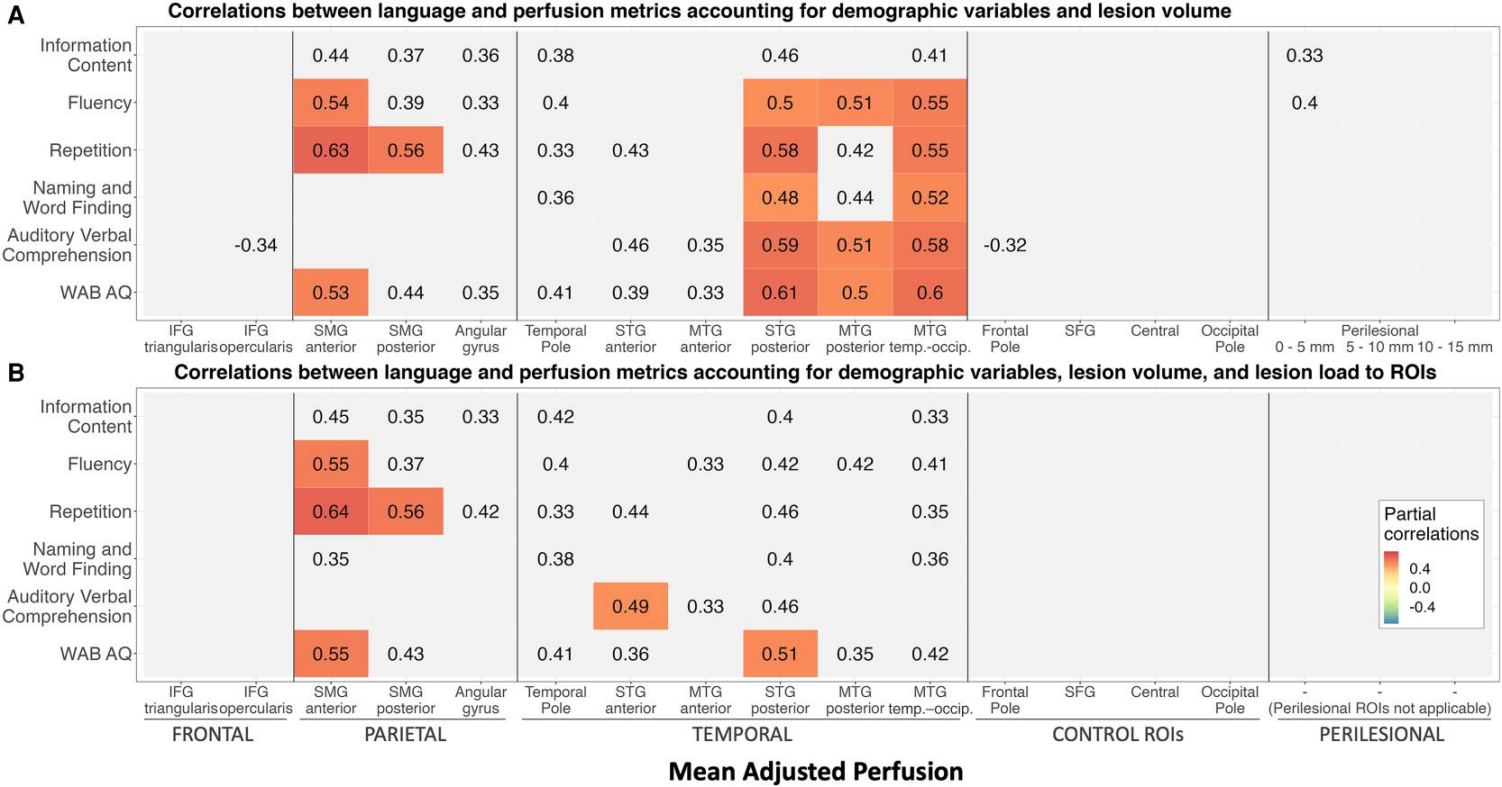
**Partial correlations between perfusion measures and language scores.** (**A**) Partial Pearson correlations in the aphasia group (*n* = 43) between language and perfusion metrics in left hemisphere ROIs accounting for age, gender, time post-onset, scanning site, and lesion volume. (**B**) Partial Pearson correlations in the aphasia group (*n* = 43) between language and perfusion metrics in left hemisphere ROIs accounting for age, gender, time post-onset, scanning site, lesion volume, and lesion load to individual ROIs. Correlations significant at *P* < 0.05 are printed, while significant correlations after adjusting for multiple comparisons (*P* < 0.0033) are colour-coded according to their values. See Supplementary Table A5 for relevant test statistics.

Partial correlations were performed between language measures and perfusion levels in different atlas-based and perilesional ROIs in the aphasia group accounting for relevant demographic (age, sex, time post-onset, and scanning site) and lesion variables (lesion volume or lesion load to a particular ROI). In Fig. 6A, correlations between language and perfusion metrics accounting for demographic variables and lesion volume are presented, while in Fig. 6B, correlations between language and perfusion metrics accounting also for lesion load to individual ROIs are shown. Note that the latter analysis is not applicable to perilesional ROIs, as perilesional bands by definition are not lesioned in any participants. No significant correlations were observed between perfusion levels in the right hemisphere ROIs and language abilities even prior to corrections for multiple comparisons. Accordingly, Fig. 6 only includes left hemisphere regions. See Supplementary Table A5 for full results of the correlation analysis.

As can be seen from Fig. 6A, correlations remained significant and passed the threshold for multiple comparisons for inferior parietal areas (SMG) and posterior temporal areas (both STG and MTG) when only lesion volume was accounted for. The analysis accounting for lesion load (Fig. 6B) after correction for multiple comparisons did not yield the same pattern of significant correlations between language measures and perfusion levels in the posterior temporal regions. The strength of the association was likely diminished as specifically in those posterior temporal areas, there was a significant relationship between lesion load and perfusion levels, as shown previously. Subsequently when lesion load was accounted for (Fig. 6B), only correlations with the SMG remained significant and limited correlations between STG and language comprehension and general aphasia severity. Further, perfusion levels in perilesional areas were only marginally related to expressive language abilities and did not survive the correction for multiple comparisons. Overall, the data show that perfusion levels in areas beyond the lesion site in the left hemisphere significantly impacted performance on subtests of the WAB even after accounting for demographic and lesion variables (lesion volume and lesion load), while perfusion in the right hemisphere was not associated with residual language abilities.

### Lasso regression

While the correlation analysis above provides important insights into the individual role of perfusion in different ROIs to residual language abilities, it does not demonstrate which regions contribute jointly to the observed outcomes and does not outline which regions are most salient for language. To investigate the joint impact of both perfusion and lesion load in different ROIs in the left hemisphere on language outcomes simultaneously, a regularized lasso regression was performed with each of the WAB subtest scores as the outcome variable. Respective regression coefficients are presented in Table 2, with higher values indicative of a stronger relationship with language measures and zero representing lack of such a relationship. Overall, the predictors included in the model accounted for a substantial amount of variance (*R*^2^ ranged from 0.5 to 0.8), except in the case of repetition where the model accounted for only 17% of the variance and only identified contributions of parietal areas. For the other language abilities, the analyses identified several ROIs in the parietal and temporal lobes simultaneously contributing to language processing, with the SMG and the posterior temporal areas being the strongest predictors. The SMG was particularly strongly related to fluency, while the posterior MTG was related to naming and auditory comprehension. Only perilesional tissue in the 0–5 mm band showed a small relationship with expressive language abilities. For the control regions, only the SFG was marginally associated with fluency. As expected, perfusion in other control areas showed no relationship with language scores.

**Table 2:** Results of lasso regression analysis with WAB subtest scores as the dependent variables and perfusion in left hemisphere ROIs along with demographic variables and lesion load in left hemisphere ROIs as predictors

	Information content	Fluency	Repetition	Naming	Auditory comprehension	WAB AQ
Covariates						
Lesion volume	−0.176	0	−0.169	0	−0.081	−0.176
Age	0	0	−0.052	0.082	0	0
Time post-onset	0.143	0	0.153	0.256	0	0.143
Scanning site	0	0	0	0.058	0	0
Gender	0	0	0	0.061	0	0
Mean adjusted perfusion						
IFG triangularis	0	0	0	0	0	0
IFG opercularis	0	0	0	0	0	0
SMG anterior	0.222	0.051	0	0.033	0.129	0.222
SMG posterior	0	0.048	0	0	0	0
Angular gyrus	0	0	0	0	0	0
Temporal pole	0.053	0	0.021	0	0	0.053
STG anterior	0	0	0	0	0	0
MTG anterior	0	0	0	0	0	0
STG posterior	0	0	0	0	0	0
MTG posterior	0.121	0	0.102	0.157	0	0.121
MTG temp.–occip.	0.06	0	0	0	0.085	0.06
Frontal pole	0	0	0	0	0	0
SFG	0.01	0	0	0	0	0.01
Central	0	0	0	0	0	0
Occipital pole	0	0	0	0	0	0
Perilesional 0–5 mm	0.129	0.118	0	0.167	0	0.058
Perilesional 5–10 mm	0	0	0	0	0	0
Perilesional 10–15 mm	0	0	0	−0.09	0	0
Lesion load						
IFG triangularis	0	0	0	0	0	0
IFG opercularis	0	0	0.184	0.121	0	0
SMG anterior	0	0	0	0	0	0
SMG posterior	−0.217	0	−0.297	−0.139	−0.203	−0.217
Angular gyrus	0	−0.047	0	0	−0.01	0
Temporal Pole	−0.009	0	−0.156	−0.055	0	−0.009
STG anterior	0	0	−0.125	0	0	0
MTG anterior	0	0	−0.023	0	0	0
STG posterior	0	0	0	−0.097	0	0
MTG posterior	0	0	−0.033	0	0	0
MTG temp.–occip.	−0.129	0	−0.127	−0.506	−0.123	−0.129
Frontal pole	−0.098	0	−0.022	0	0	−0.098
SFG	0	0	0	0	0	0
Central	0	0	0	0	0	0
Occipital pole	−0.082	0	−0.254	0	0	−0.082
**Lambda**	0.128	0.078	0.515	0.05	0.061	0.221
***R* ^2^**	0.567	0.738	0.167	0.806	0.722	0.538
**RMSE**	0.65	0.506	0.902	0.436	0.521	0.672

Regarding the lesion load covariate, we observed that greater lesion load was associated with lower language scores, particularly in perisylvian ROIs (hence, the negative coefficients). The only exception to this was the positive relationship between lesion load to the IFG opercularis and language outcomes. This pattern likely indicates that lesions specifically to that frontal area led to less severe language deficits, as critical posterior regions were spared in those instances (for a similar argument, see Zhong *et al*.^47^).

## Discussion

In the current study, we first investigated how perfusion outside of lesioned areas was affected in chronic post-stroke aphasia in comparison to perfusion in age-matched controls. We then determined how perfusion in specific cortical and perilesional areas was related to language outcomes in aphasia. To investigate perfusion in aphasia, we used anatomically defined ROIs from the Harvard–Oxford atlas that covered the frontal, parietal, and temporal areas of the perisylvian cortex in both hemispheres, along with several control regions not implicated previously in language processing. For the PWA group, we also analysed perfusion levels in tissue at different distances from the lesion. We compared perfusion levels between the PWA and control groups and investigated the relationship between perfusion levels and WAB subtest scores using both correlation and regularized regression analyses.

First, the current study demonstrated that cerebral perfusion in chronic stroke is greatly reduced even beyond the lesion site, with overall whole-brain perfusion significantly lower in the aphasia group compared to age-matched controls. The reduction in CBF is primarily noticeable in the lesioned hemisphere, with raw perfusion reduced in most regions in the left hemisphere and some regions in the contralesional hemisphere. Once ROI-specific perfusion values were normalized by the individual’s whole-brain perfusion, these adjusted perfusion levels in PWA still remained significantly reduced compared to controls in specific frontal and parietal areas outside of the lesion in the left lesioned hemisphere, while no statistically significant differences between PWA and controls were detected in homologous right hemisphere regions. This reduction in ipsilesional (left hemisphere) perfusion in areas distant from the lesion site has been observed in previous studies in both acute and chronic stroke using initially positron emission tomography and more recently MRI perfusion-weighted imaging.^5,15,16,18-20,25,48,49^ However, we did not observe a pattern of hyperperfused right hemisphere regions, as found by Thompson *et al*.^16^ Perilesional tissue closer to the lesion, as expected, showed lower perfusion compared to more distant perilesional areas, as has been documented in previous studies.^16-19^ Outside of the tissue within 5 mm of the lesion, perfusion values returned to normal and were comparable to the rest of the brain. Furthermore, perfusion levels in the left hemisphere ROIs in PWA were lower compared to homologous right hemisphere regions, confirming previous findings of interhemispheric asymmetry in aphasia.^16,25^

A similar trend of interhemispheric differences was observed for healthy controls, although to a lesser extent, and the difference between perfusion in left and right hemispheres was only significant for four ROIs. While detailed examination of the observed interhemispheric differences in healthy controls is beyond the scope of the current study, we speculate that these physiological asymmetries may reflect normal variation in perfusion, lateralization of specific cortical functions,^50^ or the potential influence of external factors.^51^ Because asymmetry in perfusion was observed in healthy controls, it is important to compare such interhemispheric asymmetries between groups to ascertain that interhemispheric differences in stroke are beyond the range of normal variation in perfusion. Accordingly, asymmetry of perfusion between homologous left and right ROIs was more pronounced in the PWA group compared to the control group for both frontal and parietal regions. This result further supports the observation that greater decline in perfusion specifically in these ipsilateral areas is present in the aphasia group.

Second, we found that perfusion in the left temporal lobe (most strongly in the posterior part of both STG and MTG) and in the inferior parietal areas (SMG) was significantly related to different WAB subtest scores. This relationship was present even when direct lesion damage to these areas was accounted for, both in the partial correlation and the regularized regression analyses. This result indicates that spared blood flow in those areas is important for supporting language function. However, even if that tissue is preserved (as evidenced by an absence of a lesion on structural scans), it may display varying levels of functionality. Suboptimal CBF can affect function via impaired neurovascular coupling. CBF might be no longer matched to the metabolic requirements of the tissue, and thus suboptimally perfused critical language areas might not be able to support residual processing. In contrast, perfusion in the frontal ROIs did not show such a relationship. This pattern is similar to a recent small study of perfusion in stroke patients, where perfusion levels—specifically in temporoparietal areas—were related to residual language abilities.^5^ Further, our findings are aligned with results seen by Thompson *et al*.,^16^ although their correlations between perfusion and language scores did not survive correction for multiple comparisons. As expected, no relationship with language was observed for perfusion levels in control areas within the ipsilesional hemisphere.

The relationship observed between CBF in temporoparietal areas and language outcomes aligns with other recent investigations highlighting the critical role that these areas play in language processing and post-stroke aphasia recovery. Traditionally these temporal areas were considered important for lexical–semantic processing,^52^ both in word retrieval,^53-55^ single-word comprehension,^55,56^ and repetition.^54,57^ Contemporary evidence also suggests that they support higher-level syntactic processing.^58^ Thus, our results outlining the critical role of temporoparietal areas in comprehension, production, and repetition align with prior studies using other brain mapping methods. The lack of frontal contributions to language outcomes might be surprising at first, but we believe it is coherent with emerging findings suggesting that frontal areas are involved in general top-down cognitive control/supervisory functions supporting language processing.^59^ In the same vein, damage to dorsolateral prefrontal areas does not lead to long-term language deficits,^60,61^ and their level of activation does not seem to significantly contribute to language recovery post-stroke.^62^ Lesion data from the current study is very much in line with these observations, as in the lasso regression, analysis lesions to the IFG opercularis were inversely associated with language outcomes. That is, greater damage specifically to frontal cortical areas led to less pronounced language deficits, most likely due to critical posterior language regions being spared in those cases (similar to Zhong *et al*.^47^). However, damage to the underlying white matter in the frontal lobes has been previously associated with long-term language production impairments.^60,63^ Further, non-invasive brain stimulation of the left IFG has been shown to have positive effects on language performance^64,65^ (see Berube and Hillis for a review^66^). These observed positive effects following stimulation of the frontal regions are not incompatible with results from lesion studies. As stated above, frontal regions might be necessary for providing domain general support (e.g. working memory resources) for language processing which requires preserved structural connections to other language areas. On the contrary, the findings of the current study specifically demonstrate that perfusion levels in the frontal cortical regions in the chronic stages of recovery do not contribute to language outcomes in post-stroke aphasia. To resolve these differences, a more comprehensive study involving multimodal neuroimaging is warranted that can quantify the differential contributions of frontal grey matter, white matter, and perfusion as well as their underlying interactions to language recovery post-stroke.

In the perilesional analysis, only perfusion in the band of 0–5 mm was marginally related to language production abilities, particularly measures of fluency, but it did not survive the correction for multiple comparisons. In the regularized regression analysis, only perilesional perfusion in the 0–5 mm band was again related to language production abilities, including fluency and naming ability, and overall aphasia severity. Thus, of all the perilesional bands that we investigated, only perfusion in tissue directly adjacent to the lesion was pertinent for language outcomes. Possibly, increased perfusion in that perilesional tissue helps to promote functional compensation for the adjoining lesion, while more distant perilesional ROIs (5–10 mm and 10–15 mm) likely encompass different cytoarchitectonic areas that are unable to compensate properly for the lesioned tissue. Furthermore, consistent with most prior investigations, no relationship between perfusion in the contralesional (right hemisphere) ROIs and language abilities was observed.^16,25^

Overall, the results of the partial correlation analyses and the regularized regression were in concordance with each other. Areas identified in the partial correlation analyses were also shown to be predictive of language outcomes in the regularized regression. The uniqueness of the regularized regression is that it shows the joint contribution of multiple areas at the same time, highlighting which areas work together to support residual language abilities. All the language abilities, apart from repetition, were well predicted with the regularized regression models. It is possible that repetition, amongst all the language abilities tested in this study, is the most focal one and also relies more heavily on white matter integrity,^63^ something that was not accounted for in the current study.

Collectively, these results argue that, as the language system reorganizes itself, the initial functional specialization of a region might be more pertinent than proximity to the lesion, with functionality of core temporoparietal language areas in the left hemisphere being most critical for determining residual language deficits. In other words, perfusion levels in language areas, rather than in perilesional tissue, determined the level of residual language abilities. Surprisingly no consistent strong associations were found between perilesional perfusion and post-stroke language abilities. This finding is likely due to the fact that participants were scanned in the chronic stage of their aphasia, when critical changes in the perilesional space have already taken place.^21,67-69^ It may also be the case that perilesional perfusion is more relevant in cases of motor recovery, as motor function is more modular and spatially restricted, i.e. more distant areas are not capable of taking on functions of the motor cortex.^70^

### Limitations

One limitation of the current study was the older parameters of the MRI perfusion sequence in the VA cohort. According to contemporary standards,^31,71^ 1800–2000 ms label duration and post-labelling delays for clinical populations are recommended since shorter durations may reflect slower blood flow dynamics in addition to decreased perfusion. To examine this possibility, a separate analysis of just the VA group was run (see Supplementary Figs B1 and B2) and demonstrated a similar pattern to the full cohort. This finding provides confidence that the reported results cannot be attributed exclusively to altered transit delays. To counter these limitations, future stroke studies should ideally use longer post-labelling delays (as used for the UCB cohort) or multiple PLD sequences where transit times can be estimated directly.^71,72^ This approach will provide a more comprehensive depiction of blood flow levels and dynamics in the stroke population.

The current cross-sectional study was also limited to exploring the role of perfusion in chronic stroke. Future investigations should explore changes in perfusion as individuals regain their language abilities in the first months post-stroke to better understand its causal and predictive role in stroke recovery, particularly in perilesional tissue and in temporoparietal regions.^73^ Additionally, while the current investigation took a significant step forward in terms of investigating the differential contributions of perfusion to language outcomes while accounting for structural cortical damage, the possible impact of structural disconnections on behavioural outcomes was not assessed. Moreover, the relationship between neural activity, connectivity, and perfusion in stroke is altered due to impaired neurovascular coupling and should be systematically examined. These questions will need to be comprehensively addressed in future multimodal neuroimaging studies.

## Conclusions

The current study represents the largest exploration of perfusion in chronic post-stroke aphasia to date.^73^ Most importantly, we comprehensively investigated the relationship between perfusion in multiple regions simultaneously and language abilities while accounting for structural cortical damage, allowing us to uncover the unique contribution of residual perfusion to language outcomes. Overall, the results demonstrate that blood flow is reduced beyond the lesion site in chronic post-stroke aphasia and hypoperfused neural tissue in critical temporoparietal language areas is not fully able to support recovery, leading to more pronounced residual language deficits. The findings underscore the critical and general role that left hemisphere posterior temporal regions play in various expressive and receptive language abilities.^52,55,61,63^ Overall, the study shows that slowed or reduced blood distribution can affect the functionality of regions beyond the lesion site and have a direct impact on behavioural outcomes.

The current study highlights the importance of exploring CBF measures in stroke. Perfusion MRI can be used alongside functional MRI in a complementary manner as it offers biologically meaningful quantitative measurement of tissue metabolism and function. Relative to task-based functional MRI, perfusion offers insights into functionality of all the brain regions simultaneously, not just those involved in the execution of a given task. Furthermore, a perfusion protocol can be easier to administer, and can be more readily standardized across sites and hence included in routine clinical assessments. Perfusion measures, while rarely implemented in multimodal neuroimaging studies^73^ (with a notable exception by Kristinsson *et al*.^74^), may offer valuable prognostic indicators of recovery potential and should be routinely included in future studies investigating the neural mechanisms of post-stroke recovery.

## Supplementary Material

fcad252_Supplementary_Data

## Data Availability

The data sets presented in this article are not readily available: research data are not shared per Department of Veteran Affairs privacy regulations. Requests to access the data sets should be directed to Juliana V. Baldo, Juliana.Baldo@va.gov.
