# Supplementary material for: Cerebral perfusion in post-stroke aphasia and its relationship to residual language abilities
Source: Brain Commun. 2023 Oct 5;6(1):fcad252. doi: 10.1093/braincomms/fcad252 (PMC10757451; doi:10.1093/braincomms/fcad252)
Supplement: fcad252_Supplementary_Data [file fcad252_supplementary_data.docx]

**Supplementary Appendix A – Descriptive statistics and results of statistical tests for the whole cohort.**

Table A1. *Descriptive statistics (Mean (SD)) of mean raw perfusion for left and right hemisphere ROIs in the aphasia and the control groups, along with results of between group comparison (aphasia vs. control group). Significant tests (p < .0033) are in bold.*

| **ROI** | **Aphasia (M (SD))** | | **Controls (M (SD))** | | **Left hemisphere comparisons** | | | **Right hemisphere comparisons** | | |
| --- | --- | --- | --- | --- | --- | --- | --- | --- | --- | --- |
|  | **LH** | **RH** | **LH** | **RH** | **Test** | **statistic** | **p-value** | **Test** | **statistic** | **p-value** |
| IFG triangularis | 25.77 (13.89) | 31.43 (11.44) | 36.36 (8.71) | 39.34 (9.96) | **Welch-test** | **-3.86** | **0.0002597** | T-test | -2.88 | 0.0053782 |
| IFG opercularis | 25.31 (13.13) | 35.29 (11.43) | 40.3 (9.66) | 42.68 (9.41) | **T-test** | **-4.96** | **0.0000054** | T-test | -2.74 | 0.0079305 |
| SMG anterior | 19.92 (9.72) | 26 (8.96) | 29.73 (7.25) | 29.73 (5.78) | **T-test** | **-4.38** | **0.0000433** | Wilcox-test | 381.00 | 0.0472330 |
| SMG posterior | 22.32 (9.73) | 29.42 (8.88) | 33.93 (6.64) | 37.34 (7.2) | **Welch-test** | **-5.83** | **0.0000002** | **T-test** | **-3.79** | **0.0003307** |
| Angular gyrus | 23.55 (10.14) | 29.67 (9.44) | 36.48 (6.75) | 38.31 (7.84) | **Wilcox-test** | **165.00** | **0.0000022** | **T-test** | **-3.87** | **0.0002553** |
| Temporal Pole | 22.93 (10.8) | 28.71 (11.7) | 32.58 (9.08) | 34.71 (10.23) | **T-test** | **-3.76** | **0.0003671** | T-test | -2.13 | 0.0367816 |
| STG anterior | 28.74 (12.92) | 36.23 (12.39) | 38.45 (8.07) | 39.83 (9.97) | **Welch-test** | **-3.76** | **0.0003723** | Wilcox-test | 446.00 | 0.2470875 |
| MTG anterior | 22.98 (13.09) | 24.62 (12.08) | 30.58 (11.29) | 31.45 (10.03) | Wilcox-test | 334.00 | 0.0135243 | T-test | -2.39 | 0.0198897 |
| STG posterior | 28.42 (10.51) | 37.5 (10.74) | 40.5 (9.63) | 48.79 (9.53) | **T-test** | **-4.67** | **0.0000158** | **Wilcox-test** | **228.00** | **0.0000849** |
| MTG posterior | 26.06 (12.34) | 32.37 (11.35) | 33.97 (8.96) | 38.66 (9.28) | T-test | -2.80 | 0.0066482 | Wilcox-test | 333.00 | 0.0094666 |
| MTG temp.-occip. | 28.13 (11.18) | 35.63 (11.55) | 37.15 (7.55) | 41.2 (8.69) | **Welch-test** | **-3.96** | **0.0001904** | Wilcox-test | 344.00 | 0.0140956 |
| Frontal Pole | 18.5 (8.81) | 22.33 (8.07) | 28.28 (7.98) | 30.45 (7.26) | **T-test** | **-4.57** | **0.0000221** | **Wilcox-test** | **231.00** | **0.0000994** |
| SFG | 22.49 (9.45) | 24.22 (8.27) | 27.82 (8.57) | 27.38 (9.43) | T-test | -2.32 | 0.0235239 | T-test | -1.44 | 0.1539328 |
| Central | 22.51 (10) | 27.54 (8.72) | 31.23 (6.12) | 33.24 (6.13) | **Welch-test** | **-4.46** | **0.0000326** | T-test | -2.88 | 0.0053929 |
| Occipital Pole | 22.15 (9.75) | 26.34 (12) | 30.3 (7.94) | 33.15 (9.04) | **Wilcox-test** | **245.00** | **0.0002040** | **Wilcox-test** | **279.00** | **0.0010323** |

Table A2. *Descriptive statistics (Mean (SD)) of mean adjusted perfusion for left and right hemisphere ROIs in the aphasia and the control groups, along with results of between group comparison (aphasia vs. control group). Significant tests (p < .0033) are in bold.*

| **ROI** | **Aphasia (M (SD))** | | **Controls (M (SD))** | | **Left hemisphere comparisons** | | | **Right hemisphere comparisons** | | |
| --- | --- | --- | --- | --- | --- | --- | --- | --- | --- | --- |
|  | **LH** | **RH** | **LH** | **RH** | **Test** | **statistic** | **p-value** | **Test** | **statistic** | **p-value** |
| IFG triangularis | 0.94 (0.35) | 1.21 (0.31) | 1.12 (0.19) | 1.21 (0.19) | Wilcox-test | 364.00 | 0.0277763 | Wilcox-test | 541.00 | 0.9695619 |
| IFG opercularis | 0.92 (0.33) | 1.37 (0.31) | 1.25 (0.21) | 1.32 (0.17) | **Welch-test** | **-4.99** | **0.0000049** | Welch-test | 0.91 | 0.3645874 |
| SMG anterior | 0.75 (0.27) | 1.01 (0.27) | 0.92 (0.14) | 0.92 (0.11) | Wilcox-test | 315.00 | 0.0047476 | Welch-test | 1.93 | 0.0579147 |
| SMG posterior | 0.84 (0.22) | 1.14 (0.22) | 1.05 (0.13) | 1.16 (0.11) | **Wilcox-test** | **208.00** | **0.0000286** | Welch-test | -0.28 | 0.7806069 |
| Angular gyrus | 0.89 (0.22) | 1.15 (0.24) | 1.13 (0.13) | 1.18 (0.1) | **Wilcox-test** | **149.00** | **0.0000008** | Welch-test | -0.83 | 0.4098371 |
| Temporal Pole | 0.88 (0.38) | 1.1 (0.31) | 1 (0.19) | 1.06 (0.22) | Wilcox-test | 386.00 | 0.0547811 | T-test | 0.50 | 0.6156078 |
| STG anterior | 1.13 (0.47) | 1.41 (0.4) | 1.2 (0.18) | 1.23 (0.24) | Welch-test | -0.75 | 0.4558823 | Welch-test | 2.29 | 0.0254819 |
| MTG anterior | 0.88 (0.41) | 0.92 (0.37) | 0.93 (0.24) | 0.96 (0.21) | Welch-test | -0.70 | 0.4848236 | Welch-test | -0.59 | 0.5594751 |
| STG posterior | 1.15 (0.42) | 1.47 (0.3) | 1.25 (0.17) | 1.52 (0.23) | Welch-test | -1.40 | 0.1670569 | T-test | -0.70 | 0.4838612 |
| MTG posterior | 1.01 (0.39) | 1.25 (0.27) | 1.05 (0.17) | 1.19 (0.16) | Wilcox-test | 485.00 | 0.5083549 | Welch-test | 1.06 | 0.2913353 |
| MTG temp.-occip. | 1.1 (0.36) | 1.38 (0.28) | 1.15 (0.13) | 1.27 (0.16) | Welch-test | -0.80 | 0.4247602 | Welch-test | 2.03 | 0.0469028 |
| Frontal Pole | 0.68 (0.17) | 0.85 (0.15) | 0.87 (0.15) | 0.94 (0.11) | **T-test** | **-4.58** | **0.0000213** | T-test | -2.46 | 0.0165881 |
| SFG | 0.85 (0.23) | 0.94 (0.21) | 0.85 (0.16) | 0.83 (0.19) | Welch-test | 0.00 | 0.9987794 | T-test | 2.06 | 0.0436407 |
| Central | 0.84 (0.22) | 1.07 (0.22) | 0.97 (0.12) | 1.03 (0.1) | **Welch-test** | **-3.11** | **0.0027797** | Welch-test | 0.94 | 0.3510641 |
| Occipital Pole | 0.83 (0.2) | 0.99 (0.22) | 0.93 (0.16) | 1.02 (0.16) | Wilcox-test | 347.00 | 0.0156637 | T-test | -0.60 | 0.5509859 |

Table A3. *Results of comparison of mean adjusted perfusion between homologous left and right hemisphere ROIs for the aphasia and the control groups separately, and comparison of asymmetry of right-left hemisphere perfusion between the aphasia and the control groups. Significant tests (p < .0033) are in bold.*

| **ROI** | **Aphasia group**  **(left-right hemisphere comparison)** | | | **Control group**  **(left-right hemisphere comparison)** | | | **Asymmetry of right-left hemisphere perfusion between groups** | | |
| --- | --- | --- | --- | --- | --- | --- | --- | --- | --- |
|  | **Test** | **statistic** | **p-value** | **Test** | **statistic** | **p-value** | **Test** | **statistic** | **p-value** |
| IFG triangularis | **Wilcox-test** | **181.00** | **0.0004318** | Wilcox-test | 100.00 | 0.0952704 | Wilcox-test | 683.00 | 0.0651405 |
| IFG opercularis | **Wilcox-test** | **46.00** | **0.0000004** | T-test | -1.80 | 0.0840421 | **Wilcox-test** | **822.00** | **0.0001211** |
| SMG anterior | **T-test** | **-5.49** | **0.0000021** | T-test | -0.26 | 0.7965654 | **Welch-test** | **4.48** | **0.0000308** |
| SMG posterior | **Wilcox-test** | **20.00** | **0.0000000** | **Wilcox-test** | **31.00** | **0.0004238** | **Wilcox-test** | **858.00** | **0.0000470** |
| Angular gyrus | **Wilcox-test** | **46.00** | **0.0000003** | Wilcox-test | 70.00 | 0.0133074 | **Wilcox-test** | **828.00** | **0.0002255** |
| Temporal Pole | **Wilcox-test** | **151.00** | **0.0001036** | T-test | -1.64 | 0.1134062 | Wilcox-test | 740.00 | 0.0101907 |
| STG anterior | **T-test** | **-3.37** | **0.0016728** | T-test | -0.88 | 0.3894343 | Welch-test | 2.57 | 0.0126106 |
| MTG anterior | T-test | -0.78 | 0.4372597 | Wilcox-test | 152.00 | 0.7878765 | Wilcox-test | 569.00 | 0.5727968 |
| STG posterior | **T-test** | **-4.72** | **0.0000288** | **T-test** | **-5.70** | **0.0000071** | Welch-test | 0.63 | 0.5331223 |
| MTG posterior | **T-test** | **-4.22** | **0.0001295** | **T-test** | **-4.16** | **0.0003500** | Welch-test | 1.40 | 0.1670073 |
| MTG temp.-occip. | **T-test** | **-5.23** | **0.0000051** | **T-test** | **-3.54** | **0.0016808** | Welch-test | 2.46 | 0.0166567 |
| Frontal Pole | **T-test** | **-6.52** | **0.0000001** | T-test | -3.22 | 0.0036829 | **Welch-test** | **3.13** | **0.0025819** |
| SFG | Wilcox-test | 261.00 | 0.0106538 | T-test | 1.15 | 0.2618524 | Wilcox-test | 738.00 | 0.0109638 |
| Central | **Wilcox-test** | **75.00** | **0.0000016** | **T-test** | **-3.37** | **0.0025425** | **Wilcox-test** | **818.00** | **0.0003689** |
| Occipital Pole | **T-test** | **-4.17** | **0.0001479** | T-test | -2.46 | 0.0214251 | T-test | 1.32 | 0.1899395 |

Table A4. *Descriptive statistics (Mean (SD)) of adjusted perfusion for the left and right hemisphere ROIs in the aphasia group with (‘Lesioned ROI’) and without a lesion (‘Spared ROI’) in a given ROI, along with results of between group comparison (‘Lesioned ROI’ vs. ‘Spared ROI’ group). Significant tests (p < .0033) are in bold.*

| **ROI** | **Left hemisphere (M(SD))** | | **Right hemisphere (M(SD))** | | **Left hemisphere comparisons** | | | **Right hemisphere comparisons** | | |
| --- | --- | --- | --- | --- | --- | --- | --- | --- | --- | --- |
|  | **Lesioned** | **Spared** | **Lesioned** | **Spared** | **Test** | **statistic** | **p-value** | **Test** | **statistic** | **p-value** |
| IFG triangularis | 0.78 (0.39) | 1.01 (0.31) | 1.13  (0.2) | 1.24 (0.34) | Wilcox-test | 276.00 | 0.0602371 | Wilcox-test | 256.00 | 0.1736103 |
| IFG opercularis | 0.71 (0.27) | 1.05 (0.29) | 1.35 (0.31) | 1.38 (0.32) | **T-test** | **3.86** | **0.0004062** | T-test | 0.36 | 0.7221218 |
| SMG anterior | 0.68 (0.31) | 0.79 (0.24) | 1.02 (0.31) | 1.01 (0.25) | T-test | 1.38 | 0.1739264 | T-test | -0.11 | 0.9115537 |
| SMG posterior | 0.76 (0.25) | 0.9 (0.18) | 1.12 (0.21) | 1.17 (0.24) | T-test | 2.26 | 0.0294068 | T-test | 0.72 | 0.4779021 |
| Angular gyrus | 0.83  (0.3) | 0.93 (0.15) | 1.12 (0.22) | 1.17 (0.25) | Wilcox-test | 267.00 | 0.2044909 | T-test | 0.71 | 0.4789955 |
| Temporal Pole | 0.87 (0.47) | 0.89 (0.32) | 1.14 (0.35) | 1.07 (0.29) | Wilcox-test | 247.00 | 0.5264579 | T-test | -0.63 | 0.5336456 |
| STG anterior | 1.16  (0.6) | 1.12 (0.42) | 1.53 (0.31) | 1.35 (0.42) | T-test | -0.21 | 0.8342946 | T-test | -1.43 | 0.1605239 |
| MTG anterior | 0.62  (0.2) | 0.96 (0.43) | 0.89 (0.28) | 0.93 (0.4) | **Welch-test** | **3.43** | **0.0016168** | T-test | 0.34 | 0.7392967 |
| STG posterior | 0.84 (0.34) | 1.26 (0.4) | 1.51 (0.31) | 1.45 (0.31) | T-test | 3.05 | 0.0041134 | Wilcox-test | 179.00 | 0.6818892 |
| MTG posterior | 0.76 (0.32) | 1.14 (0.36) | 1.21 (0.28) | 1.27 (0.27) | **T-test** | **3.45** | **0.0013221** | T-test | 0.61 | 0.5484176 |
| MTG temp.-occip. | 0.81 (0.32) | 1.24 (0.29) | 1.27 (0.23) | 1.43 (0.29) | **T-test** | **4.45** | **0.0000653** | T-test | 1.82 | 0.0760549 |
| Frontal Pole | 0.59 (0.15) | 0.72 (0.17) | 0.86 (0.13) | 0.85 (0.15) | T-test | 2.34 | 0.0243701 | T-test | -0.27 | 0.7909259 |
| SFG | 0.86 (0.39) | 0.85 (0.19) | 1.11 (0.15) | 0.9 (0.21) | Welch-test | -0.09 | 0.9315408 | T-test | -2.79 | 0.0079612 |
| Central | 0.79 (0.19) | 0.9 (0.23) | 1.06 (0.22) | 1.07 (0.22) | T-test | 1.74 | 0.0885192 | T-test | 0.10 | 0.9194859 |
| Occipital Pole | 0.82 (0.17) | 0.83 (0.21) | 1.16 (0.19) | 0.96 (0.21) | Wilcox-test | 116.00 | 0.7546400 | T-test | -2.35 | 0.0238074 |

Table A5. *Partial Pearson correlations (r (p-value)) between language and perfusion metrics in left hemisphere ROIs: Part A – accounting for age, gender, time post-onset, scanning site, and lesion volume; Part B – additionally accounting for lesion load to individual ROIs. Significant tests (p < .0033) are in bold.*

|  | **IFG triangularis** | **IFG opercularis** | **SMG anterior** | **SMG posterior** | **Angular gyrus** | **Temporal Pole** | **STG anterior** | **MTG anterior** | | **STG posterior** | **MTG posterior** | **MTG temp.-occip.** | **Frontal Pole** | **SFG** | **Central** | **Occipital Pole** | **Perilesional**  **0-5 mm** | **Perilesional**  **5-10 mm** | **Perilesional**  **10-15 mm** |
| --- | --- | --- | --- | --- | --- | --- | --- | --- | --- | --- | --- | --- | --- | --- | --- | --- | --- | --- | --- |
|  | **Part A – Partial correlations accounting for demographic variables and lesion volume** | | | | | | | | | | | | | | | | | | |
| **Information Content** | 0.009 (.957) | -0.051 (.765) | 0.439 (.006) | 0.37 (.022) | 0.359 (.027) | 0.382 (.018) | 0.246 (.148) | 0.273 (.102) | 0.459 (.005) | | 0.319 (.051) | 0.405 (.012) | -0.03 (.857) | 0.071 (.671) | 0.105 (.532) | 0.06 (.722) | 0.325 (.046) | 0.219 (.188) | 0.056 (.738) |
| **Fluency** | 0.082 (.625) | -0.044 (.795) | **0.541 (<.001)** | 0.387 (.016) | 0.327 (.045) | 0.4 (.013) | 0.229 (.18) | 0.295 (.076) | **0.497 (.002)** | | **0.511 (.001)** | **0.547 (<.001)** | -0.036 (.829) | 0.077 (.648) | 0.177 (.289) | 0.044 (.793) | 0.404 (.012) | 0.286 (.081) | 0.131 (.432) |
| **Repetition** | -0.097 (.561) | -0.197 (.244) | **0.635 (<.001)** | **0.561 (<.001)** | 0.432 (.007) | 0.328 (.044) | 0.43 (.009) | 0.204 (.225) | **0.585 (<.001)** | | 0.422 (.008) | **0.553 (<.001)** | -0.236 (.153) | 0.059 (.725) | 0.142 (.396) | -0.093 (.579) | 0.264 (.109) | 0.129 (.442) | -0.013 (.939) |
| **Naming and Word Finding** | -0.093 (.577) | -0.158 (.35) | 0.314 (.055) | 0.236 (.154) | 0.172 (.303) | 0.358 (.027) | 0.315 (.062) | 0.324 (.05) | **0.479 (.003)** | | 0.439 (.006) | **0.517 (.001)** | -0.218 (.188) | -0.107 (.523) | -0.069 (.681) | 0.063 (.709) | 0.25 (.13) | 0.165 (.323) | -0.077 (.647) |
| **Auditory Verbal Comprehension** | -0.19 (.252) | -0.341 (.039) | 0.304 (.063) | 0.286 (.082) | 0.17 (.307) | 0.274 (.095) | 0.461 (.005) | 0.355 (.031) | **0.587 (<.001)** | | **0.515 (.001)** | **0.576 (<.001)** | -0.323 (.048) | -0.193 (.245) | -0.133 (.426) | 0.017 (.921) | 0.024 (.885) | 0.034 (.841) | -0.098 (.559) |
| **WAB AQ** | -0.061 (.718) | -0.173 (.306) | **0.531 (.001)** | 0.436 (.006) | 0.348 (.032) | 0.407 (.011) | 0.388 (.019) | 0.332 (.044) | **0.607 (<.001)** | | **0.505 (.001)** | **0.599 (<.001)** | -0.19 (.254) | -0.009 (.959) | 0.065 (.699) | 0.016 (.922) | 0.31 (.058) | 0.2 (.229) | 0.004 (.982) |
|  | **Part B – Partial correlations accounting for demographic variables, lesion volume, and lesion load to individual ROIs** | | | | | | | | | | | | | | | | | | |
| **Information Content** | 0.123 (.467) | 0.199 (.244) | 0.448 (.005) | 0.351 (.033) | 0.335 (.043) | 0.417 (.01) | 0.158 (.366) | 0.188 (.273) | | 0.396 (.018) | 0.201 (.233) | 0.333 (.044) | 0.048 (.78) | 0.007 (.968) | 0.034 (.841) | -0.037 (.829) | na | na | na |
| **Fluency** | 0.196 (.245) | 0.176 (.303) | **0.553 (<.001)** | 0.369 (.024) | 0.298 (.073) | 0.397 (.015) | 0.287 (.094) | 0.334 (.047) | | 0.422 (.011) | 0.422 (.009) | 0.41 (.012) | 0.013 (.941) | 0.018 (.918) | 0.121 (.475) | -0.035 (.836) | na | na | na |
| **Repetition** | -0.023 (.891) | -0.011 (.951) | **0.64 (<.001)** | **0.564 (<.001)** | 0.417 (.01) | 0.326 (.049) | 0.436 (.009) | 0.17 (.323) | | 0.463 (.005) | 0.266 (.112) | 0.353 (.032) | -0.159 (.349) | -0.067 (.695) | 0.061 (.721) | -0.128 (.451) | na | na | na |
| **Naming and Word Finding** | -0.005 (.977) | 0.063 (.715) | 0.352 (.033) | 0.201 (.234) | 0.125 (.463) | 0.376 (.022) | 0.218 (.208) | 0.257 (.13) | | 0.402 (.017) | 0.28 (.093) | 0.36 (.029) | -0.138 (.416) | -0.157 (.353) | -0.132 (.434) | -0.06 (.724) | na | na | na |
| **Auditory Verbal Comprehension** | -0.137 (.42) | -0.218 (.202) | 0.323 (.051) | 0.259 (.122) | 0.104 (.54) | 0.274 (.101) | **0.491 (.003)** | 0.333 (.047) | | 0.459 (.006) | 0.319 (.054) | 0.255 (.128) | -0.251 (.134) | -0.232 (.167) | -0.196 (.245) | 0.009 (.957) | na | na | na |
| **WAB AQ** | 0.044 (.794) | 0.069 (.69) | **0.551 (<.001)** | 0.431 (.008) | 0.319 (.054) | 0.413 (.011) | 0.364 (.032) | 0.291 (.085) | | **0.51 (.002)** | 0.353 (.032) | 0.419 (.01) | -0.106 (.531) | -0.092 (.587) | -0.016 (.926) | -0.067 (.693) | na | na | na |

**Supplementary Appendix B – Figures, descriptive statistics, and results of statistical tests for the VA cohort only.**

**
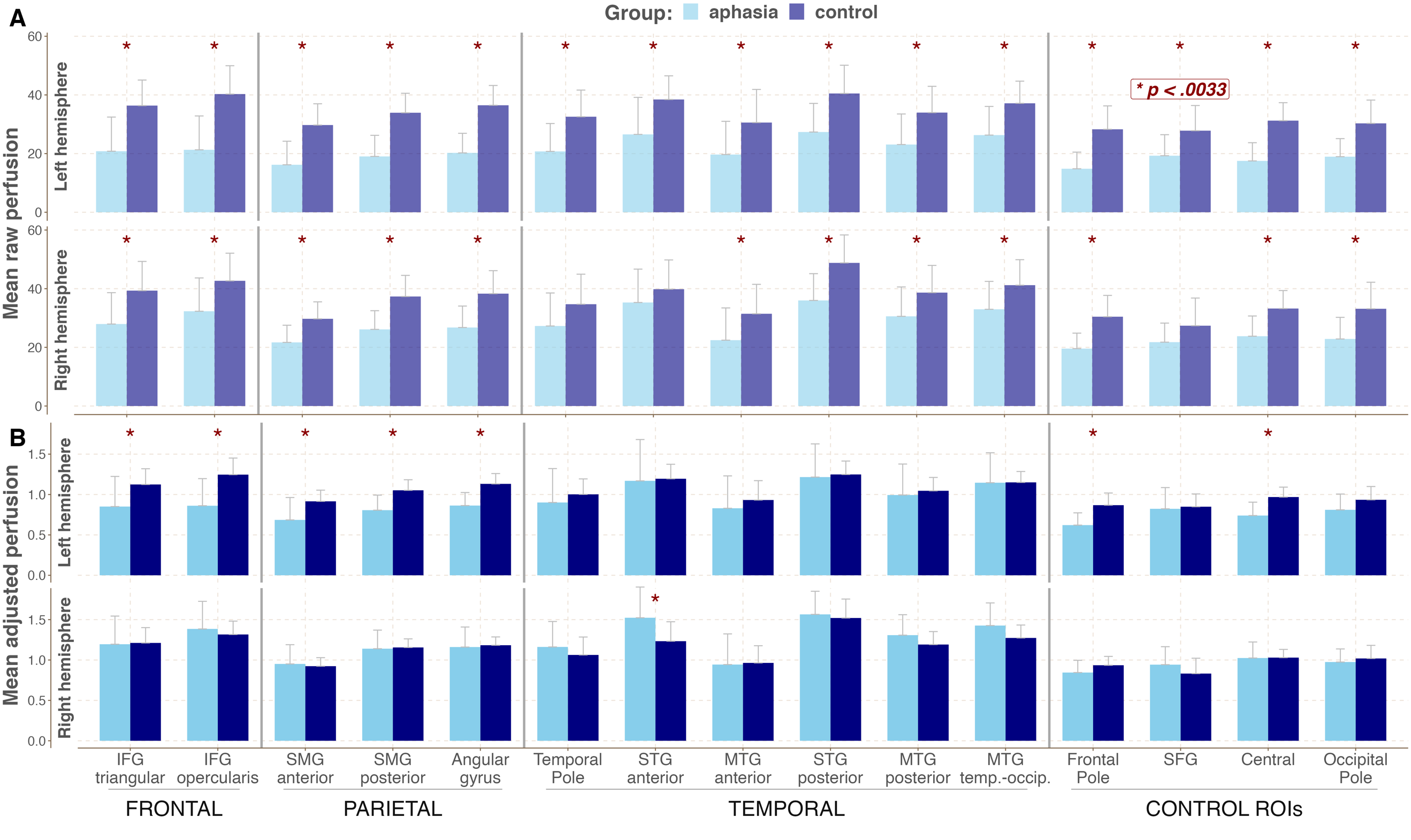
**

**Figure B1.** Mean perfusion values for the aphasia and the control groups from the VA cohort only across left and right hemisphere ROIs. Red asterisks mark significant differences between groups for a given ROI. Panel A – Mean raw perfusion. Panel B – Mean adjusted perfusion. IFG – inferior frontal gyrus, SMG – supramarginal gyrus, STG – superior temporal gyrus, MTG – middle temporal gyrus, temp.-occip. – temporal-occipital, SFG – superior frontal gyrus, ROIs – regions of interest.


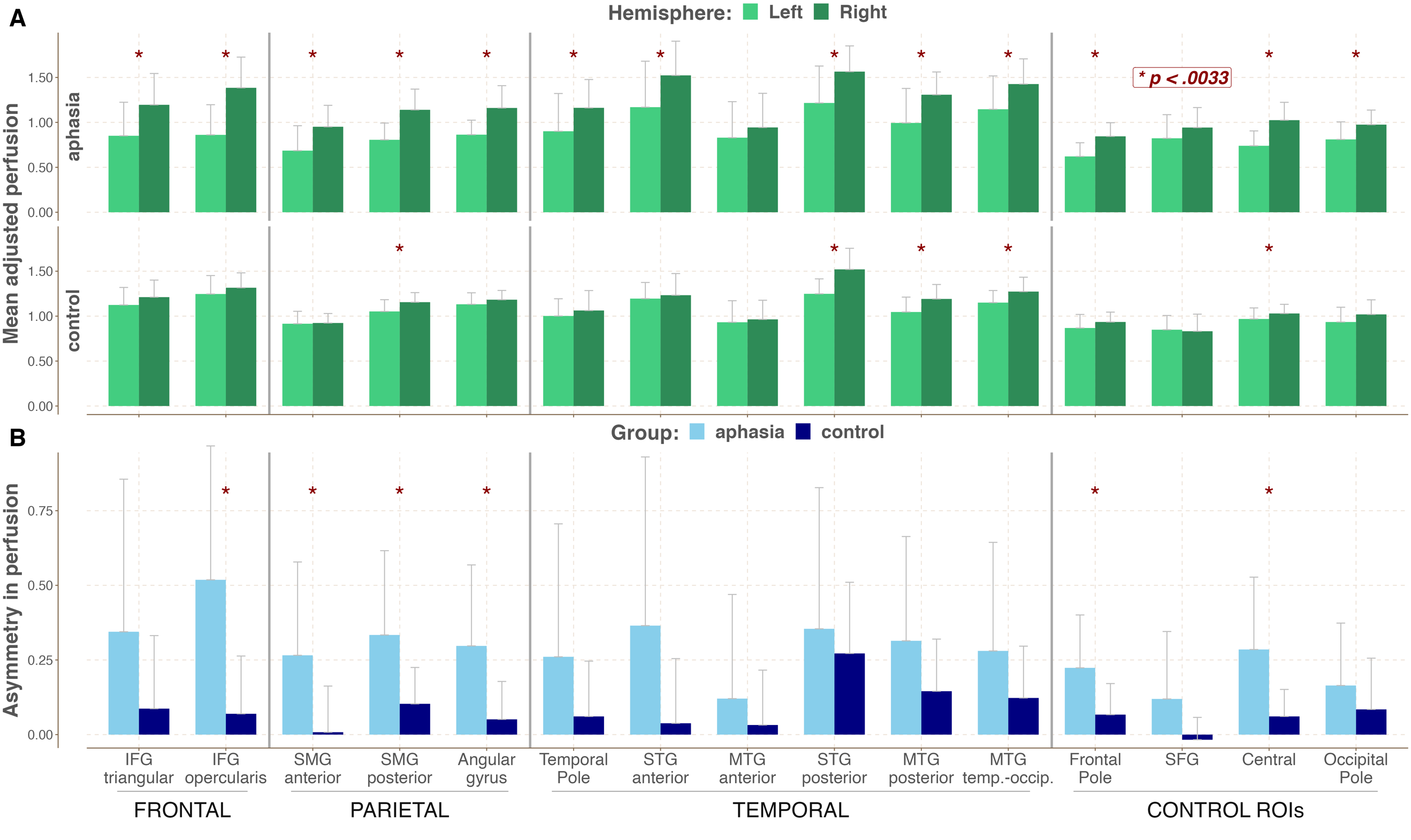


**Figure B2.** Comparison of perfusion in the left and right hemisphere ROIs between the two groups from the VA cohort only­. Red asterisks mark significant differences. *Panel A* – Mean adjusted perfusion for the left and right hemisphere ROIs in the aphasia and the control groups. *Panel B* – Asymmetry of right-left hemisphere perfusion between the aphasia and the controls groups. IFG – inferior frontal gyrus, SMG – supramarginal gyrus, STG – superior temporal gyrus, MTG – middle temporal gyrus, temp.-occip. – temporal-occipital, SFG – superior frontal gyrus, ROIs – regions of interest.

Table B1. *Descriptive statistics (Mean (SD)) of mean adjusted perfusion for left and right hemisphere ROIs in the VA and UCB aphasia cohorts separately, along with results of between group comparison (VA cohort vs. UCB cohort). Significant tests (p < .0033) are in bold.*

| **ROI** | **VA cohort**  **(M (SD))** | | **UCB cohort**  **(M (SD))** | | **Left hemisphere comparisons between cohorts** | | | **Right hemisphere comparisons between cohorts** | | |
| --- | --- | --- | --- | --- | --- | --- | --- | --- | --- | --- |
|  | **LH** | **RH** | **LH** | **RH** | **Test** | **statistic** | **p-value** | **Test** | **statistic** | **p-value** |
| IFG triangularis | 0.85 (0.37) | 1.2 (0.35) | 1.12 (0.21) | 1.23 (0.2) | Welch-test | -3.01 | 0.0044826 | Wilcox-test | 222.00 | 0.6315952 |
| IFG opercularis | 0.86 (0.34) | 1.38 (0.34) | 1.05 (0.28) | 1.34 (0.24) | T-test | -1.77 | 0.0842358 | T-test | 0.48 | 0.6351534 |
| SMG anterior | 0.69 (0.28) | 0.95 (0.24) | 0.88 (0.21) | 1.14 (0.3) | Wilcox-test | 123.00 | 0.0393527 | T-test | -2.26 | 0.0288964 |
| SMG posterior | 0.81 (0.19) | 1.14 (0.23) | 0.92 (0.28) | 1.15 (0.22) | T-test | -1.56 | 0.1273909 | T-test | -0.18 | 0.8603583 |
| Angular gyrus | 0.86 (0.16) | 1.16 (0.25) | 0.94 (0.31) | 1.12 (0.21) | Wilcox-test | 153.00 | 0.1995130 | T-test | 0.47 | 0.6414121 |
| Temporal Pole | 0.9 (0.42) | 1.16 (0.32) | 0.84 (0.3) | 0.97 (0.27) | Wilcox-test | 208.00 | 0.9071525 | T-test | 1.98 | 0.0547877 |
| STG anterior | 1.17 (0.51) | 1.52 (0.38) | 1.07 (0.39) | 1.17 (0.32) | T-test | 0.67 | 0.5070466 | T-test | 2.95 | 0.0051623 |
| MTG anterior | 0.83 (0.4) | 0.94 (0.38) | 0.97 (0.44) | 0.88 (0.35) | T-test | -1.02 | 0.3150312 | T-test | 0.53 | 0.5968550 |
| STG posterior | 1.22 (0.41) | 1.56 (0.29) | 1.01 (0.42) | 1.28 (0.25) | T-test | 1.52 | 0.1372610 | **T-test** | **3.24** | **0.0023489** |
| MTG posterior | 0.99 (0.38) | 1.31 (0.25) | 1.04 (0.41) | 1.12 (0.27) | T-test | -0.37 | 0.7138649 | T-test | 2.21 | 0.0330103 |
| MTG temp.-occip. | 1.15 (0.37) | 1.43 (0.28) | 1.01 (0.33) | 1.29 (0.26) | T-test | 1.18 | 0.2448198 | T-test | 1.58 | 0.1213757 |
| Frontal Pole | 0.62 (0.15) | 0.84 (0.15) | 0.8 (0.14) | 0.87 (0.13) | **T-test** | **-3.78** | **0.0004976** | T-test | -0.52 | 0.6084447 |
| SFG | 0.82 (0.26) | 0.94 (0.22) | 0.9 (0.15) | 0.93 (0.2) | Welch-test | -1.25 | 0.2179865 | T-test | 0.21 | 0.8355954 |
| Central | 0.74 (0.17) | 1.02 (0.2) | 1.05 (0.16) | 1.15 (0.24) | **T-test** | **-5.81** | **0.0000008** | T-test | -1.85 | 0.0712577 |
| Occipital Pole | 0.81 (0.2) | 0.98 (0.16) | 0.87 (0.22) | 1.02 (0.3) | Wilcox-test | 174.00 | 0.4601119 | Welch-test | -0.51 | 0.6172434 |
